# Characterization and functional analysis of the *BAM* gene family in peanut (*Arachis hypogaea* L.)

**DOI:** 10.3389/fpls.2025.1599610

**Published:** 2025-06-19

**Authors:** Chaonan Shi, Peilin Li, Long Li, Zhitao Qi, Tong Lu, Jinming Shi, Haodong Yang, Jin Wu, Jingyu Guo, Minghui Liu, Xiaozong Wu

**Affiliations:** ^1^ Key Laboratory of Biotechnology in Tobacco Industry, College of Tobacco Science and Engineering, Zhengzhou University of Light Industry, Zhengzhou, China; ^2^ State Key Laboratory of North China Crop Improvement and Regulation, North China Key Laboratory for Crop Germplasm Resources, College of Agronomy, Hebei Agricultural University, Hebei, Baoding, China

**Keywords:** peanut (*Arachis hypogaea* L.), β-amylase, gene family, bioinformatics analysis, expression pattern

## Abstract

β-Amylase (BAM) is a kind of amylase in plants and microorganisms, which plays an important role in regulating plant growth and development and stress response. This study conducted a genome-wide identification and analysis of the BAM gene family in peanuts, identifying a total of 18 *AhBAM* genes. The encoded proteins exhibited significant variations in length, molecular weight, and isoelectric points, with primary localization in chloroplasts and nuclei. These genes were unevenly distributed across 10 chromosomes, with chr05 and chr15 each containing three genes. Phylogenetic analysis classified them into four subfamilies, with motif 3 serving as a conserved domain, and segmental duplication identified as the primary mechanism for family expansion. Synteny analysis indicated a closer evolutionary relationship between cultivated peanuts and soybeans. Cis-acting element analysis revealed that *AhBAM* genes may participate in light signaling, hormone regulation, and stress responses. *AhBAM3* emerged as a key node within the protein-protein interaction network, then the GO analysis pinpointed starch metabolism and drought response as the primary functional enrichments for this gene family. Expression profiling showed that *AhBAM8* was highly expressed in multiple tissues, whereas most members exhibited no significant response to web blotch disease. This comprehensive analysis provides a holistic view of the potential functions of the *AhBAM* families in peanuts and lays the foundation for future experimental validation of their roles in enhancing peanut stress resistance and productivity.

## Introduction

As an important allotetraploid oil and food crop, peanut (*Arachis hypogaea* L.) is rich in plant nutrients such as isoflavones, phenolic acids, and phytosterols, and is primarily used in oil and food processing (Toomer, 2018; Zhang et al., 2024). Peanuts not only contain a large amount of unsaturated fats but also are a source of plant proteins and dietary fiber, playing a significant role in human health (Toomer, 2018). In recent years, with the successive deciphering of the genomes of peanut diploid wild species and allotetraploid cultivated species (Lu et al., 2024; Wang et al., 2025), the functional genomics of peanuts has made remarkable progress. In particular, the construction of the peanut three-dimensional genome chromatin organization map has been achieved. combined with joint analyses of Hi-C, ATAC-seq, and multi-tissue expression profiles, it has revealed the important regulatory role of chromatin spatial structure in peanut plant architecture (Zhang et al., 2021). These findings have provided new perspectives and theoretical foundations for a deeper understanding of the genetic characteristics of peanuts and their applications in agricultural production. Gene families, as collections of functionally related genes in the genome, are the core basis for plant adaptation to the environment, evolution, and developmental regulation. A deep analysis of the composition, structure, and function of peanut gene families will not only help reveal their unique biological characteristics but also provide theoretical support for the genetic improvement and sustainable development of peanuts.

Starch is not only the primary storage carbohydrate in plants, but also plays a crucial role in plant responses to abiotic stresses, such as drought, salt stress, and extreme high temperatures (Chen et al., 2022). β-Amylase (BAM) is a type of amylase widely found in plants and microorganisms, capable of catalyzing the hydrolysis of starch to produce maltose and other oligosaccharides. In plants, the activity of BAM is significantly influenced by external environmental factors such as drought, cold, and high temperatures (Monroe and Storm, 2018). In recent years, the *BAM* genes have been identified to possess diverse biological functions in various plant species, including *Arabidopsis*, maize, and cotton, and the signaling pathways and transcriptional regulatory networks in which they are involved are gradually being elucidated (David et al., 2022; Ravenburg et al., 2022; Yang et al., 2023). Although the *BAM* genes exhibit a certain degree of conservation across different plant species, significant differences in expression patterns and functions still exist among species due to interspecific variations.

In upland cotton (*Gossypium hirsutum*), 27 *GhBAM* genes have been identified, with *GhBAM7* implicated in regulating fiber strength during development (Yang et al., 2023). In addition, in maize (*Zea mays*), *ZmBAM8* demonstrates significant induction under drought, osmotic stress, and abscisic acid treatment, exhibiting chloroplast-localized activity that aligns with starch metabolism dynamics. The recombinant *ZmBAM8* protein shows high starch-hydrolyzing efficiency, and its overexpression enhances starch degradation and drought tolerance (Niu et al., 2024). Except drought responses, *BAMs* participate in cold stress adaptation in rice (*Oryza sativa*), OsMYB30 transcriptionally represses *BAM* genes, modulating starch-derived maltose accumulation to influence cold tolerance (Lv et al., 2017). Similarly, in pomegranate (*Punica granatum*), eight *PgBAM* genes contribute to pericarp development and cold stress response, with *PgBAM4* regulated by the cold-induced transcription factor *PgCBF7* (Liu et al., 2024). Jujube (*Ziziphus jujuba*) possessed nine *ZjBAM* genes, four of which (*ZjBAM1/2/5/6*) are drought-responsive and interact with α-amylase and glucanotransferase (Ma et al., 2022). These findings highlight the conserved yet specialized roles of *BAMs* in stress adaptation and developmental processes, and these studies not only provide important insights into the functional mechanisms of the *BAM* gene family but also offer potential targets for improving stress resistance and quality traits in crops through genetic engineering, holding significant theoretical and practical value.

In summary, the *BAM* gene family plays an important role in plant growth, development, and stress responses. However, the specific functions of *BAM* genes in peanut have not yet been studied. Based on this, the current study identified and characterized the peanut *BAM* gene family at the whole-genome level. Using bioinformatics approaches, we analyzed the gene structure, conserved motifs, cis-acting elements, and three-dimensional structures of peanut *BAM* family members. Moreover, by integrating transcriptome data, we further explored the potential biological functions of *BAM* family members. This study provides a theoretical basis for understanding the molecular mechanisms underlying peanut growth, development, and stress resistance and offers important targets for the development of new peanut varieties.

## Materials and methods

### Plant material and treatments

The phytohormone treatments were performed using distinct peanut cultivars - Baihua 15 for methyl jasmonate (MeJA) and Yuanza 9102 for salicylic acid (SA) - in the controlled environment of Zhengzhou University of Light Industry’s plant growth greenhouse under an 8h light/16h dark photoperiod. For each treatment, 0.5 mM hormone solutions were carefully applied to both leaf surfaces of uniformly-grown plants using standardized spraying techniques. Tissue sampling followed precise temporal patterns: MeJA-treated leaves were collected at 0h, 4h, and 12h post-application, while SA-treated samples were taken at 0h, 4h, 12h, and 24h intervals. All harvested leaf tissues underwent immediate liquid nitrogen flash-freezing followed by thorough grinding for total RNA extraction. After quality verification, high-quality RNA served as template for cDNA synthesis using the PrimeScript RT reagent kit, with the resulting cDNA subsequently employed for quantitative real-time PCR (qRT-PCR) validation analyses conducted in triplicate biological replicates. A total of seven candidate genes were validated for their expression patterns under MeJA and SA treatments using qRT-PCR analysis. Primers were shown in **Supplementary Table S1**.

### Identification and chromosomal localization of peanut *BAM* family members

The genome data of peanut were obtained from the peanut genome database (https://www.peanutbase.org/). A hidden Markov model of the typical BAM family protein structure was downloaded from the Pfam database (http://pfam.xfam.org) (Mistry et al., 2021), and search for the BAM domain (PF01373) through the HMMER 3.1 software to initially obtain the members of the peanut *BAM* family (Krejci et al., 2016). The candidate proteins identified by the aforementioned methods were submitted to the SMART (http://smart.embl-heidelberg.de) and NCBI CDD (https://www.ncbi.nlm.nih.gov/cdd/) databases for manual identification and screening to ascertain the ultimate members of the peanut *BAM* family (Lu et al., 2020; Letunic et al., 2021). Finally, 18 *AhBAM* genes were obtained. The BioPerl tool was used to predict protein physicochemical parameters (Stajich, 2007). Subcellular localization predictions were generated with WoLF PSORT (https://wolfpsort.hgc.jp/) (Horton et al., 2007). Subsequently, the locations of 18 *AhBAM* genes on chromosomes were obtained based on the information annotated for the peanut genome and analyzed through the Gene Location Visualization of TBtools (Chen et al., 2023a).

### Phylogenetic analysis, gene structure analysis and conserved motif analysis

The phylogenetic trees of peanut and its two ancestral species (*Arachis duranensis, Arachis ipaensis*), along with those of peanut, *Arabidopsis* and soybean, were respectively constructed by using the maximum likelihood method via IQTREE (Lam-Tung et al., 2015). The obtained evolutionary tree was submitted to the online website ITOL (https://itol.embl.de/) for beautification (Letunic and Bork, 2021). And the gene structure information of *BAM* family members was extracted from the peanut genome annotation file. The MEME online website (http://meme-suite.org) was used to predict the conserved domains in *AhBAM* protein sequences (Bailey et al., 2015). For this analysis, the number of motifs to be identified was set to 10, while default settings were adopted for other parameters and the results were visualized using TBtools.

### Gene duplication, and synteny analysis

*Arachis duranensis, Arachis ipaensis*, *Arabidopsis* and soybean downloaded from NCBI. To investigate the evolutionary patterns of *AhBAM* genes, we conducted comprehensive synteny analysis using MCscanX with the following analytical pipeline: First, the software identified microsynteny blocks through rigorous BLASTP alignments, enabling precise detection of both segmental and tandem duplication events within the *AhBAM* gene family. Subsequently, the algorithm established macrosyntenic relationships between peanut and its evolutionary relatives (*Arabidopsis thaliana* and *Glycine max*) by comparing conserved gene collinearity across genomes. For intuitive visualization of these complex genomic relationships, we employed Circos to generate integrated circular plots where: (i) distinct color schemes differentiated individual chromosomes, (ii) gracefully curved connectors highlighted orthologous gene pairs among species, and (iii) multi-layer heatmaps simultaneously displayed quantitative data including gene expression profiles and duplication classifications (Krzywinski et al., 2009; Wang et al., 2012).

Subsequently, to evaluate the evolutionary selection pressures acting on the duplicated *AhBAM* gene pairs, we employed KaKs Calculator 2.0 (Wang et al., 2010) for detailed sequence divergence analysis. This sophisticated computational tool implements multiple nucleotide substitution models (including NG, LWL, and YN methods) to accurately calculate: (1) synonymous substitution rates (Ks); and (2) nonsynonymous substitution rates (Ka). This analysis provided critical insights into the evolutionary forces shaping the functional diversification of *AhBAM* genes following duplication events.

### Cis−acting element analysis of *AhBAM* genes

The 2000-bp upstream promoter regions of the *AhBAM* genes were extracted using TBtools software and analyzed for cis-regulatory elements through the PlantCARE online platform (https://bioinformatics.psb.ugent.be/webtools/plantcare/html/) (Lescot et al., 2002). First, TBtools was employed to precisely isolate regulatory regions from -2000 to -1 bp relative to the transcription start site (TSS) of each *AhBAM* gene. The obtained FASTA-formatted sequences were then submitted to the PlantCARE database, then utilizing Position Weight Matrix (PWM) algorithms to identify key cis-regulatory elements such as light-responsive elements (e.g., G-box), and hormone-responsive elements (e.g., ABRE). Finally, manual verification was performed to screen prediction reliability, then the TBtools software was used to visualize the cis-regulatory elements.

### Protein−protein interaction network construction

The obtained *AhBAM* protein sequences were constructed using string database (https://string-db.org/) with the following parameters: organism set to *Arachis hypogaea*, medium confidence score (0.400), and full network type. The resulting protein−protein interaction (PPI) network was downloaded as a TSV file and imported into Cytoscape (v3.10.3) for advanced visualization, where node size represented interaction degree and edge thickness indicated confidence scores (Shannon et al., 2003; Szklarczyk et al., 2023).

### Gene Ontology analysis of *AhBAM* genes

The complete peanut protein sequence were analyzed using EGGNOG-MAPPER (http://eggnog-mapper.embl.de/) with default parameters to generate to generate comprehensive functional annotation files, including COG/KOG classifications and potential orthologous groups (Huerta-Cepas et al., 2019). For GO enrichment analysis of *AhBAM* genes, we employed TBtools with Fisher’s exact test (FDR<0.05) to identify significantly enriched terms across three ontologies: biological process, molecular function, and cellular component. The enrichment results were then visualized through the Weishengxin online platform (https://www.bioinformatics.com.cn/) for visualization (Tang et al., 2023).

### Prediction of phosphorylation, acetylation and methylation sites

The *AhBAM* protein sequence was submitted to the GPS 6.0 (https://gps.biocuckoo.cn/) for prediction of phosphorylation, acetylation and methylation modification sites (Chen et al., 2023b). To reduce the impact of false positives on the prediction results, the threshold was set to high. However, under the high threshold condition, the prediction results of methylation sites were too few. Therefore, the prediction threshold of methylation sites was set to medium. The prediction results were manually screened according to the score to obtain the final results. Visualize the prediction results using the IBS 2.0 (https://ibs.renlab.org/#/home) (Xie et al., 2022).

### Three-dimensional and secondary structure prediction

We utilized the SWISS-MODEL (https://swissmodel.expasy.org/) website to construct the three-dimensional structure model of the target protein by aligning the target protein sequence with solved protein structure templates based on known protein structure information (Waterhouse et al., 2018). We further utilized the GOR4 (https://npsa-prabi.ibcp.fr/cgi-bin/npsa_automat.pl?page=npsa_gor4.html) to predict the secondary structure of the target protein, and Its principle is based on statistical analysis and pattern recognition, by analyzing the amino acid sequence patterns in known protein structures.

### Expression patterns of *AhBAM* genes and qRT-PCR Validation

We conducted a comprehensive transcriptomic analysis of AhBAM genes using peanut genomic resources, examining both tissue-specific expression and stress responses. From the Peanut Genome Database, we analyzed RNA-seq data across eight tissues (root, stem, leaf, flower, peg, pod, and early/mature seeds) (Clevenger et al., 2016). Then analyzed RNA-seq data of peanut web blotch infection (Wu, 2024).

## Results

### Genome-wide identification and chromosomal distribution of the *BAM* gene family

A total of 18 *AhBAM* genes were identified and characterized from the peanut genome. According to their chromosomal positions, these genes were sequentially renamed from *AhBAM1* to *AhBAM18* (**Table 1**). The proteins encoded by these genes exhibit diverse lengths, with amino acid counts ranging from 229 residues in *AhBAM7* to 1144 residues in *AhBAM5*. Their relative molecular weights (MW) vary from 25,224.6 Da (*AhBAM7*) to 128,503.9 Da (*AhBAM5*), and their theoretical isoelectric points (pI) range from 5.48 (*AhBAM11*) to 9.17 (*AhBAM1*). Subcellular localization analysis revealed that the majority of AhBAM proteins are targeted to the chloroplast and nucleus, with seven members each. Two members are localized to the peroxisome, while the extracellular matrix and endoplasmic reticulum each contain one *AhBAM* gene. Based on these findings, we speculate that *AhBAM* family members may primarily performed their functions within the nucleus and chloroplast, where they could play key roles in regulating gene expression and metabolic processes.

**Table 1:** The information of *BAM* family members in peanut.

Gene Name	Gene ID	Chromosome localization	Amino acid length(aa)	MW(Da)	PI	Subcellular localization
*AhBAM1*	arahy.3VMA9Y	chr01	575	64652	9.17	Chloroplast
*AhBAM2*	arahy.NQ88PQ	chr03	559	62965.2	6.16	Chloroplast
*AhBAM3*	arahy.U96YPL	chr03	577	65146.7	8.72	Chloroplast
*AhBAM4*	arahy.I36EUT	chr04	533	58374.6	6.51	Nucleus
*AhBAM5*	arahy.UANN5Y	chr04	1144	128503.9	6.21	Nucleus
*AhBAM6*	arahy.K0N155	chr05	647	73093.8	6.44	Nucleus
*AhBAM7*	arahy.68PBWJ	chr05	229	25224.6	7.16	Nucleus
*AhBAM8*	arahy.4Y2508	chr05	585	64583.7	6.7	Peroxisome
*AhBAM9*	arahy.C3I2PC	chr08	586	66246	7.77	Chloroplast
*AhBAM10*	arahy.34GL3D	chr11	544	60998.7	8.72	Chloroplast
*AhBAM11*	arahy.0V89VS	chr13	468	52658.7	5.48	Extracellular matrix
*AhBAM12*	arahy.ABG1ZW	chr13	559	63106.3	8.45	Chloroplast
*AhBAM13*	arahy.60DV95	chr14	533	58330.5	6.36	Nucleus
*AhBAM14*	arahy.M409IX	chr14	1109	124435	5.99	Nucleus
*AhBAM15*	arahy.Y0TSMC	chr15	647	73093.8	6.44	Nucleus
*AhBAM16*	arahy.JJTS0M	chr15	482	53301.1	6.68	Endoplasmic reticulum
*AhBAM17*	arahy.TP5BL7	chr15	585	64664.7	6.45	Peroxisome
*AhBAM18*	arahy.Y6Z03A	chr18	606	68605.8	7.36	Chloroplast

**Figure 1:**
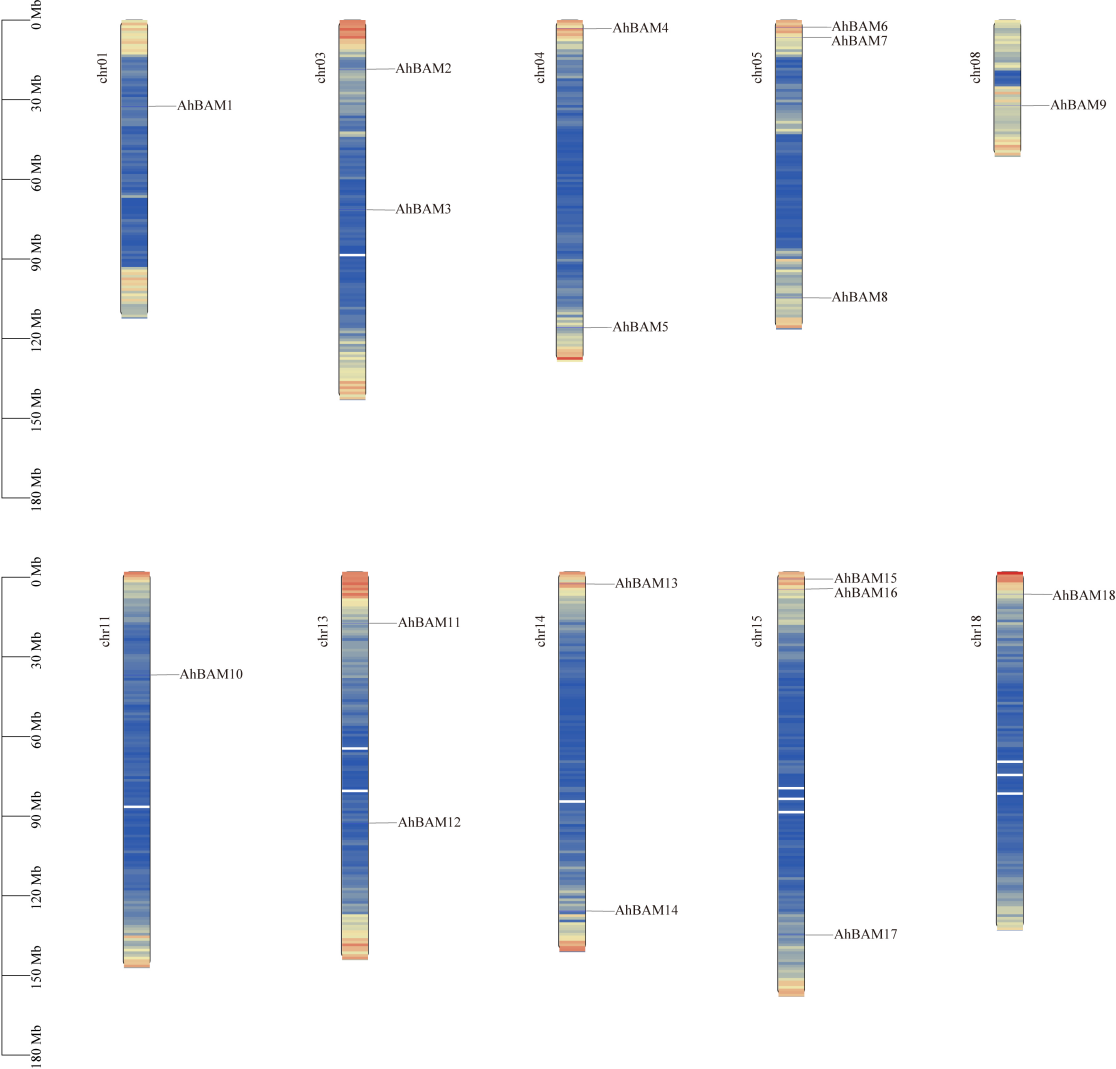
Chromosomal locations of *AhBAM* genes. The scale on the left presents the length of chromosomes (Mb).

The chromosomal localization analysis revealed that the *AhBAM* genes are unevenly distributed across 10 chromosomes in the peanut genome. Specifically, chromosomes 5 (chr05) and 15 (chr15) harbor the highest number of *AhBAM* genes, each containing three genes. Chromosomes 3 (chr03), 4 (chr04), 13 (chr13), and 14 (chr14) each possess two *AhBAM* genes, while chromosomes 1 (chr01), 8 (chr08), 11 (chr11), and 18 (chr18) each contain only a single *AhBAM* gene (**Figure 1**). This uneven distribution pattern suggests potential functional divergence and evolutionary dynamics of the *AhBAM* gene family within the peanut genome.

### Phylogenetic, gene structural, and conserved motif analyses

**Figure 2:**
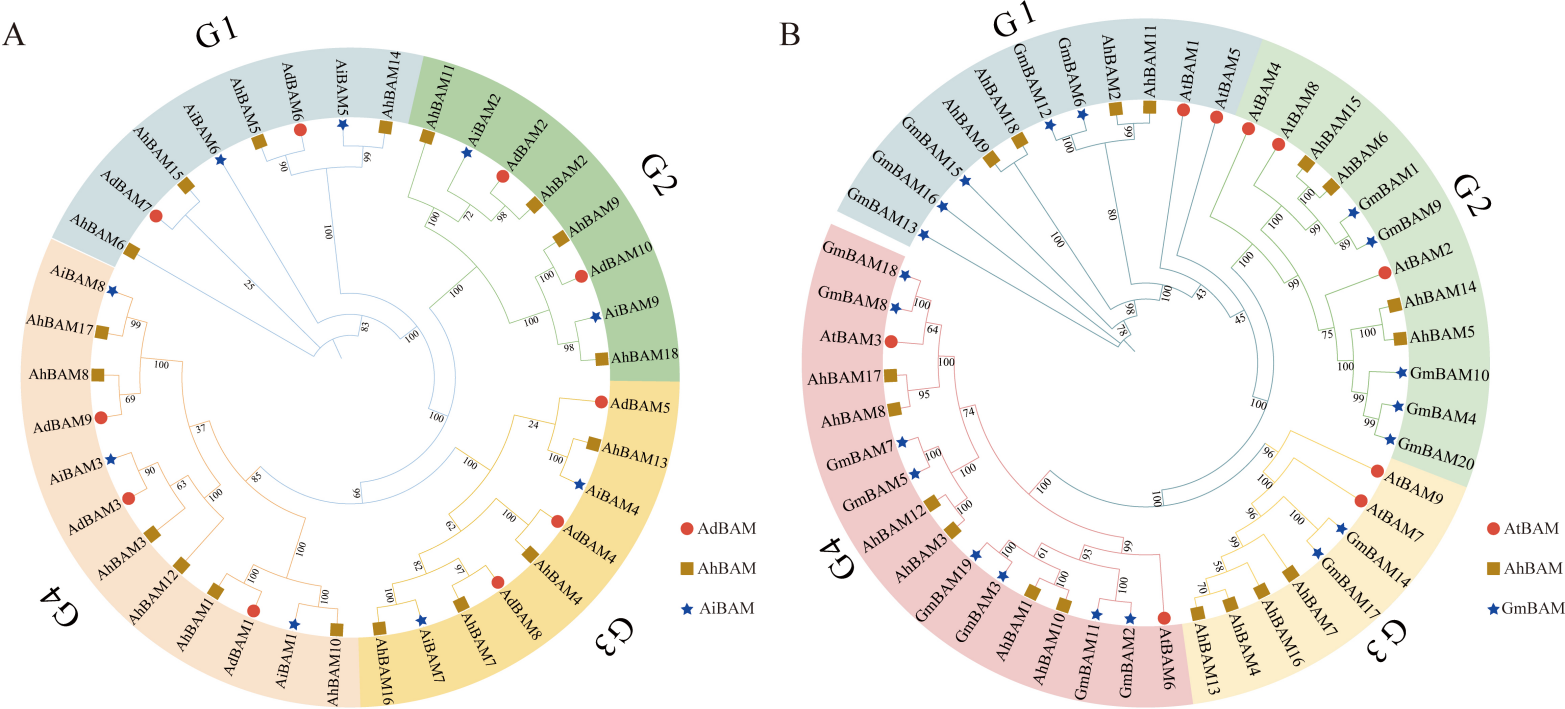
Phylogenetic analysis of the BAM family members. **(A)** Phylogenetic trees of BAM proteins for peanut, *Arachis duranensis* and *Arachis ipaensis*. **(B)** Phylogenetic trees of BAM proteins for peanut, *Arabidopsis* and soybean.

The protein sequences of the 18 identified AhBAM genes were used to construct phylogenetic trees in conjunction with those from two ancestral species (AdBAM and AiBAM), as well as from Arabidopsis (AtBAM) and soybean (GmBAM) (**Figures 2A, B**). The analysis revealed that the AhBAM genes are classified into four distinct subfamilies. Notably, the largest subfamily comprises six members of the peanut BAM gene family, while the remaining three subfamilies each contain four members.

**Figure 3:**
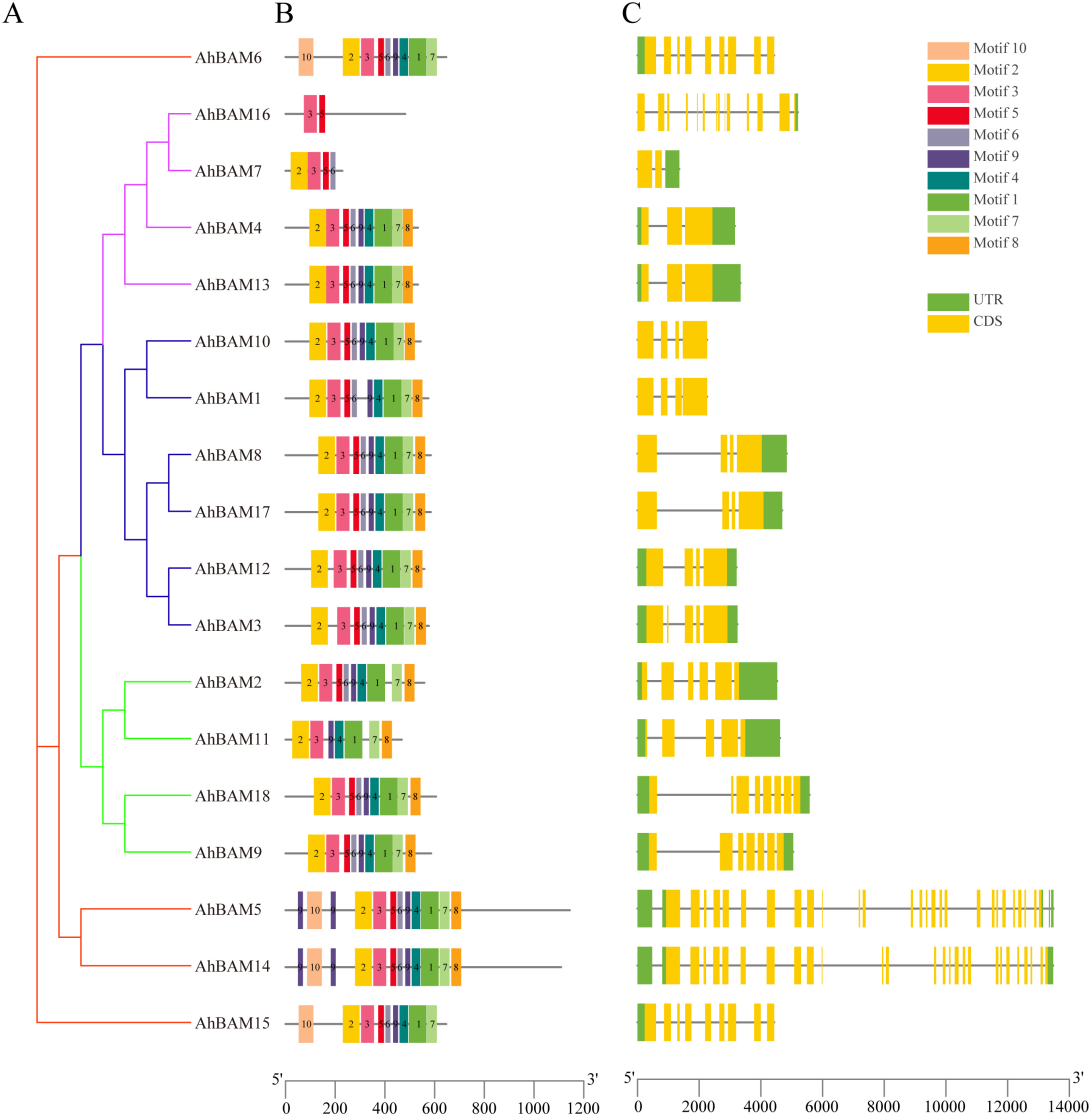
Phylogenetic tree, conserved motif, and gene structure of the *AhBAM* family in peanut. **(A)** Phylogenetic relationships of *AhBAM*. Different subgroups were marked with different colors. **(B)** Conserved motifs for *AhBAM* proteins in peanut. Different motifs are showed with different colored boxes and numbers (1–10). **(C)** The genetic structure of the *AhBAM* gene, including introns (black line), exons (yellow rectangle), and untranslated regions (UTRs, green rectangle). The scale bar of bottom demonstrates the length of exons and introns.

The conserved motif analysis within the *AhBAM* gene family revealed that Motif 3 is present in all members and represents a typical conserved domain of the *AhBAM* family. Additionally, 17 members contain both Motif 2 and Motif 5, while 16 members conclude Motif 1, Motif 4, Motif 6, Motif 7, and Motif 9. Among all *AhBAM* members, *AhBAM5* and *AhBAM14* possess all identified motifs, whereas *AhBAM16* contains only two motifs (**Figures 3A, B**). Members of the same subfamily exhibit similar gene structures. For instance, G1 subfamily members uniformly possess a higher number of introns, with *AhBAM5* and *AhBAM14* having the most (**Figure 3C**). This pattern suggests that *AhBAM* genes within the same subfamily generally share similar conserved motifs and gene structures, likely reflecting functional and evolutionary conservation within these groups.

### Gene duplication and synteny analysis

**Figure 4:**
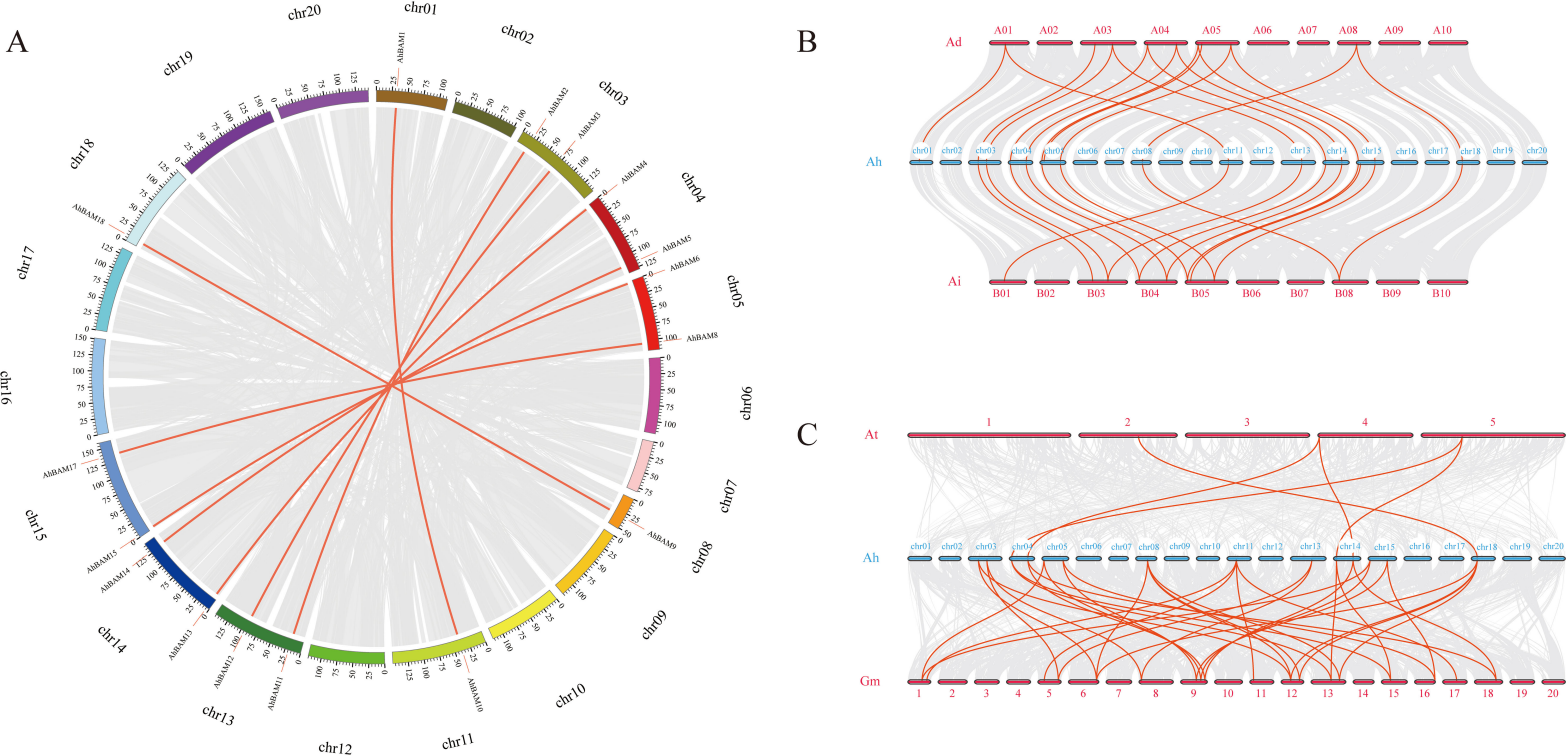
Gene duplication events in the *BAM* family. **(A)** Distribution and collinearity of the *AhBAM* gene family in the peanut genome. The gray background lines represent a collinear background and the red lines indicate a collinear relationship between *AhBAM* members. **(B)** Syntenic analysis of *BAM* genes among peanut, *Arachis duranensis* and *Arachis ipaensis.* **(C)** Syntenic analysis of *BAM* genes among peanut, *Arabidopsis* and soybean.

Among the peanut *BAM* family members, a total of eight pairs of large fragment duplicated genes were identified: *AhBAM11*: *AhBAM2*, *AhBAM10*: *AhBAM1*, *AhBAM8*: *AhBAM17*, *AhBAM13*: *AhBAM4*, *AhBAM12*: *AhBAM3*, *AhBAM9*: *AhBAM18*, *AhBAM6*: *AhBAM15*, and *AhBAM14*: *AhBAM5* (**Figure 4A**). This finding suggests that segmental duplication has been a primary mechanism driving the expansion of the peanut *BAM* gene family. Calculation of the nonsynonymous (Ka) and synonymous (Ks) substitution rates for each duplicated gene pair revealed that the Ka/Ks ratios of these *AhBAM* family members ranged from 0.063 to 0.392 (**Table 2**). These low Ka/Ks ratios indicate that these genes have undergone strong purifying selection, suggesting functional conservation and constraint during their evolutionary history.

**Table 2:** Ka/Ks of orthologous gene pairs.

Orthologous gene pairs	Ka	Ks	Ka/Ks
*AhBAM11*/*AhBAM2*	0.007	0.041	0.173
*AhBAM10*/*AhBAM1*	0.002	0.037	0.063
*AhBAM8*/*AhBAM17*	0.008	0.039	0.219
*AhBAM13*/*AhBAM4*	0.009	0.026	0.336
*AhBAM12*/*AhBAM3*	0.014	0.035	0.392
*AhBAM9*/*AhBAM18*	0.009	0.035	0.274
*AhBAM6*/*AhBAM15*	NA	NA	NA
*AhBAM14*/*AhBAM5*	0.006	0.030	0.199

The collinearity analysis among different species revealed that 16 pairs of homologous gene pairs were identified between cultivated peanuts and *Arachis duranensis*, while 15 pairs were identified between cultivated peanuts and *Arachis ipaensis* (**Figure 4B**). Additionally, comparisons between cultivated peanuts and other species showed that five homologous gene pairs were identified with *Arabidopsis thaliana*, and 34 pairs were identified with Glycine max (**Figure 4C**). These findings suggest that cultivated peanuts share a closer evolutionary relationship with soybeans, with many homologous gene pairs likely predating the divergence of their ancestral lineages. This highlights the potential conservation of key genetic elements across these species, which may have contributed to their shared adaptive traits and functional characteristics.

### Analysis of cis-acting elements in *AhBAM* genes

**Figure 5:**
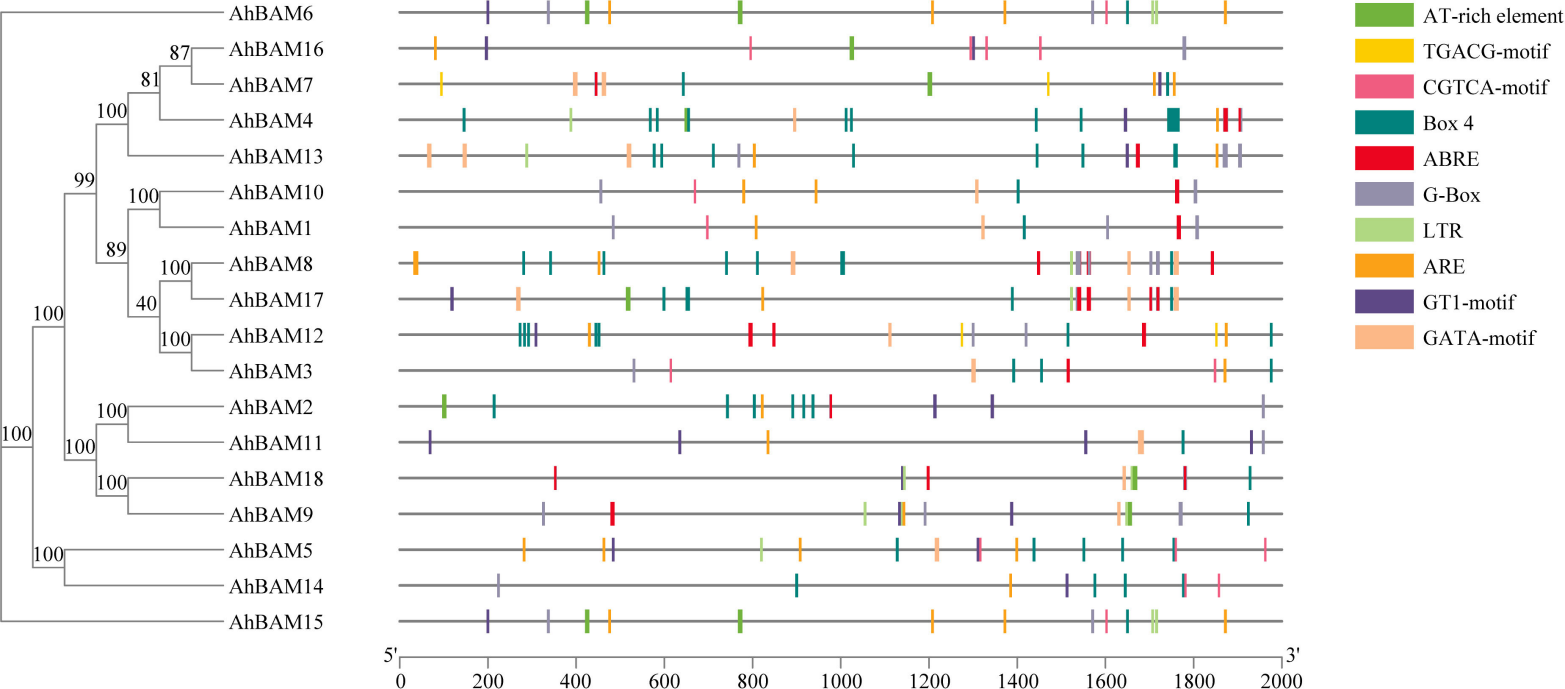
Distribution of cis-acting elements identified in the 2000 bp upstream promoter region of the peanut *AhBAM* gene.

The cis-acting elements within gene promoters directly reflect the potential functions of the corresponding genes. To elucidate the regulatory mechanisms of *AhBAM* genes, we analyzed the genomic sequences of the upstream regions (2000 base pairs) of these genes in the peanut genome. We identified and visualized 10 abundant cis-acting elements (**Figure 5**). Our analysis revealed that the promoters of *AhBAM* genes contain three main types of cis-acting elements: those involved in development, abiotic stress response, light response, and hormone response. Specifically, light-responsive elements include Box4, GATA-motif, G-Box, and GT1-motif; hormone-responsive elements include ABRE, CGTCA-motif, and TGACG-motif; and the LTR element is associated with abiotic stress response. These findings suggest that *AhBAM* genes may primarily function in light signaling, hormone regulation, and abiotic stress response pathways in peanut plants.

### Protein-protein interaction network analysis and visualization

**Figure 6:**
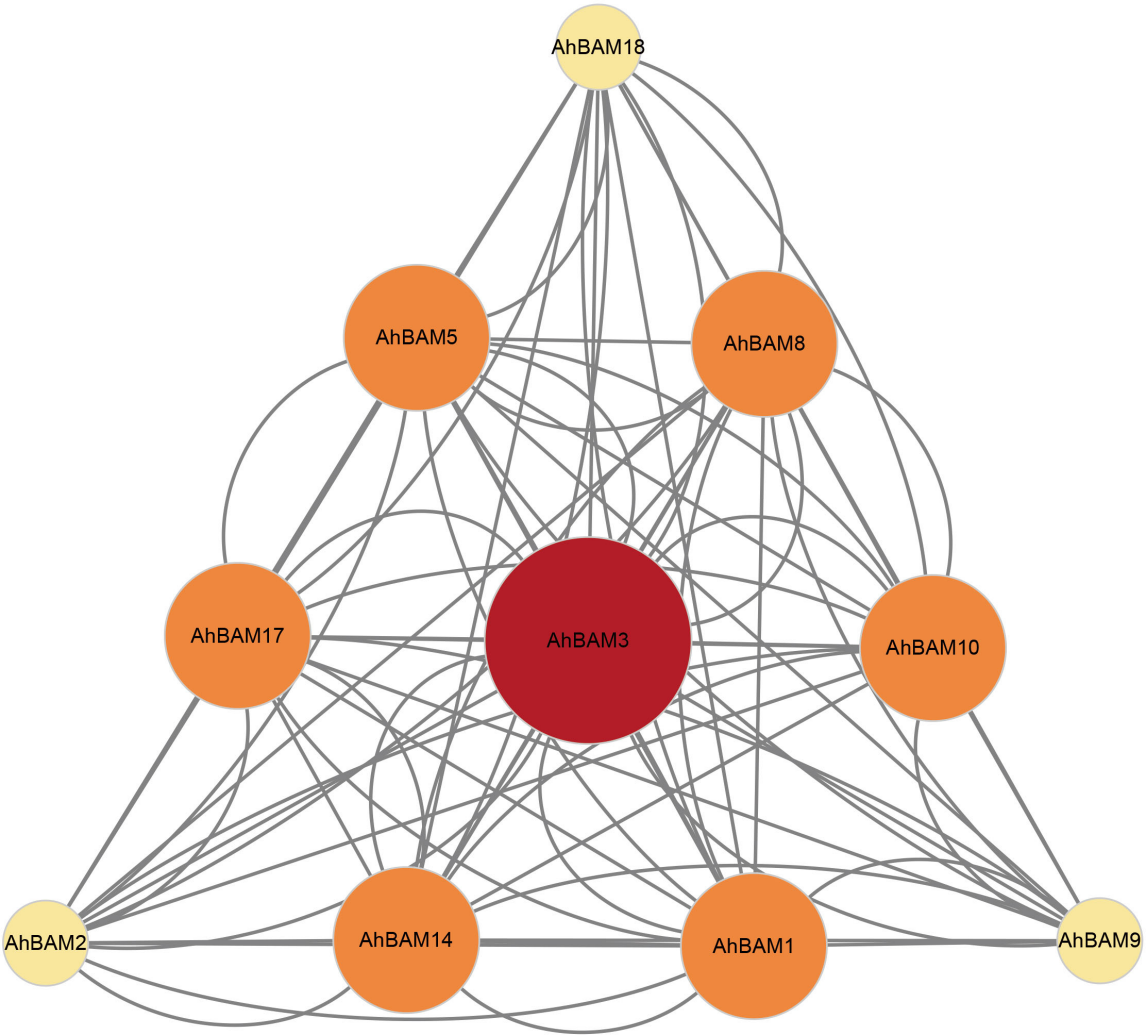
Protein–protein interaction (PPI) network of significant genes in peanut. Nodes represent proteins, central nodes are indicated in red, and black lines indicate interactions between nodes. The darker the color, the more important the protein in the interaction network.

We constructed a protein-protein interaction (PPI) network, identifying 10 AhBAM proteins that formed 13 nodes connected by 39 distinct interaction edges (**Figure 6**). These interactions reveal a complex network of connections, highlighting the complex regulatory roles of these proteins. Notably, AhBAM3 emerged as the central hub within the network, connecting to nine other genes. The hub status of AhBAM3 strongly indicates its function as a central regulator, coordinating the activity of other AhBAM proteins and serving as a potential integration node for multiple signaling pathways and regulatory mechanisms.

### GO analysis of *AhBAM* genes

**Figure 7:**
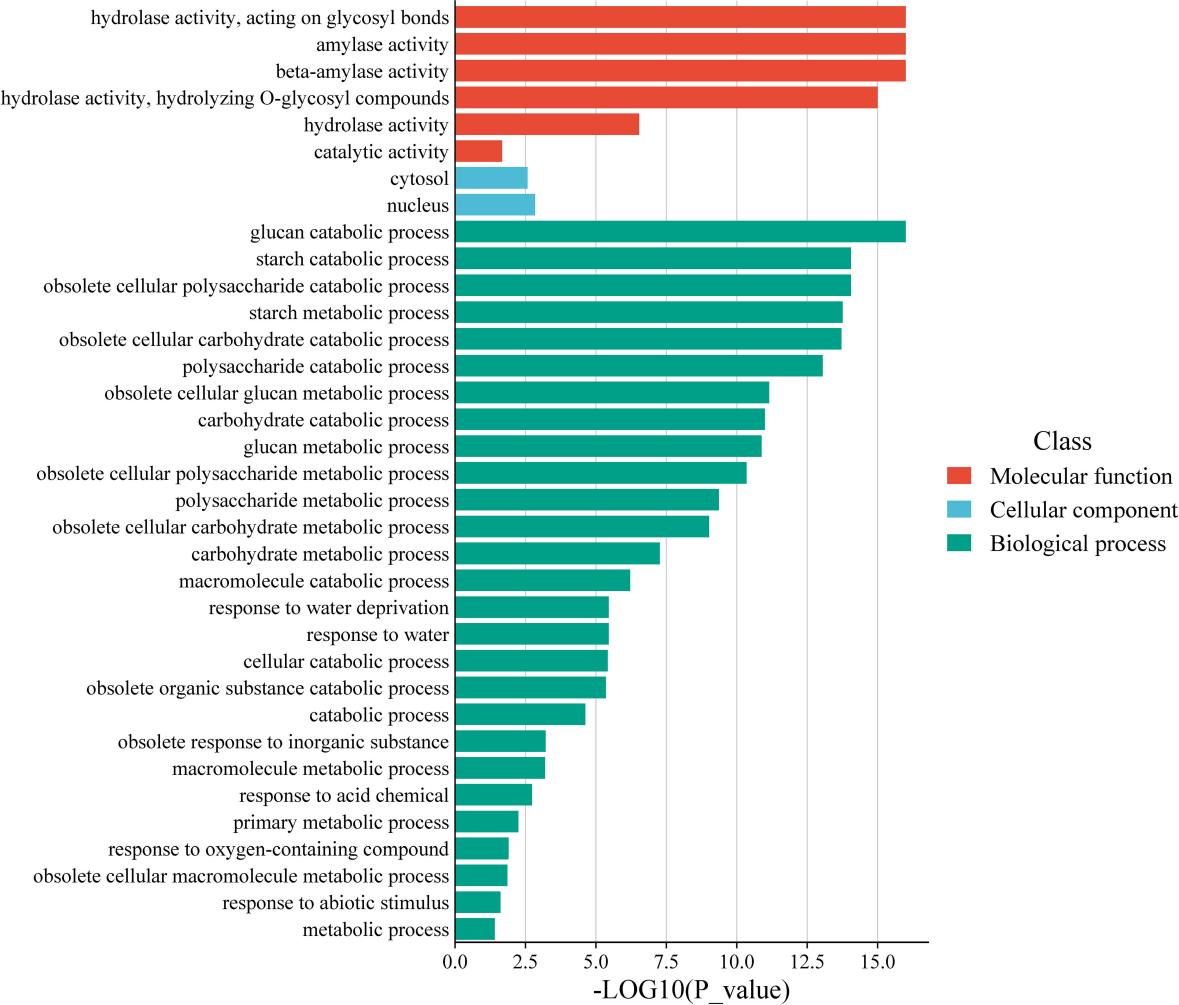
Histogram of *AhBAM* genes GO enrichment. The horizontal coordinate indicates the corrected p value, and the vertical coordinate indicates the GO term.

Gene Ontology (GO) analysis of *AhBAM* genes revealed significant enrichment in specific functional categories (**Figure 7**). At the molecular function level, *AhBAM* genes are predominantly associated with amylase activity, consistent with their role in carbohydrate metabolism. In biological processes, these genes are highly enriched in starch catabolism and glucose catabolism, further supporting their involvement in energy mobilization and metabolic homeostasis. Additionally, *AhBAM* genes also showed significant enrichment in responses to water deprivation and cellular water homeostasis, suggesting that *AhBAM* genes may play a crucial role in mediating plant responses to drought adaptation and stress-related physiological regulation. These findings indicates that *AhBAM* genes may contribute to plant survival under water-limited conditions by modulating metabolic and stress-responsive pathways.

### Prediction of post-translational modification sites: phosphorylation, acetylation, and methylation

**Figure 8:**
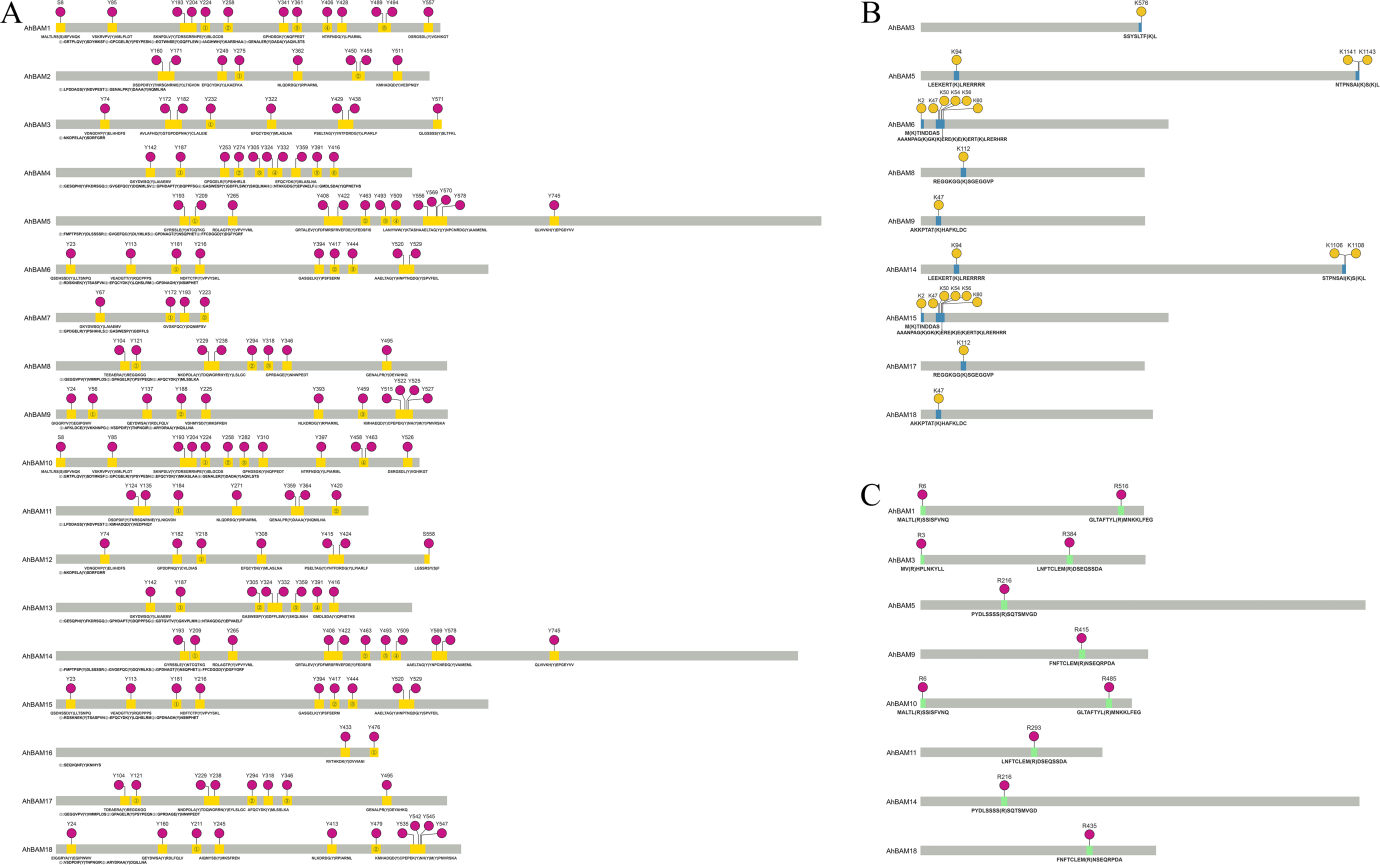
Prediction of phosphorylation, acetylation and methylation sites. **(A)** Phosphorylation site prediction of *AhBAM* gene family proteins. Red solid circle indicates the predicted phosphorylation sites, and yellow region indicates the peptide segments where the sites are located. **(B)** Acetylation site prediction. Yellow highlights the predicted acetylation sites, while blue marks the peptide segments containing these sites. **(C)** Methylation Site Prediction. Red highlights the predicted methylation sites, while green marks the corresponding peptide segments.

Bioinformatic analysis predicted multiple post-translational modification sites (PTMs) in AhBAM family proteins, including phosphorylation, acetylation, and methylation. Then tyrosine phosphorylation sites were particularly abundant within these proteins (**Figure 8A**). Among them, AhBAM1 and AhBAM5 contained the highest number (13) of phosphorylation sites, while AhBAM16 showed the fewest (2). Acetylation site prediction identified nine AhBAM proteins as potential targets for this modification (**Figure 8B**). Acetylation, a critical post-translational modification, can significantly modulate protein structure and function, these modifications may play key regulatory roles in various cellular processes. Additionally, methylation sites analysis revealed eight AhBAM proteins as likely substrates for methylation (**Figure 8C**), indicating additional regulatory complexity.

### Prediction of three-dimensional and secondary structures

**Figure 9:**
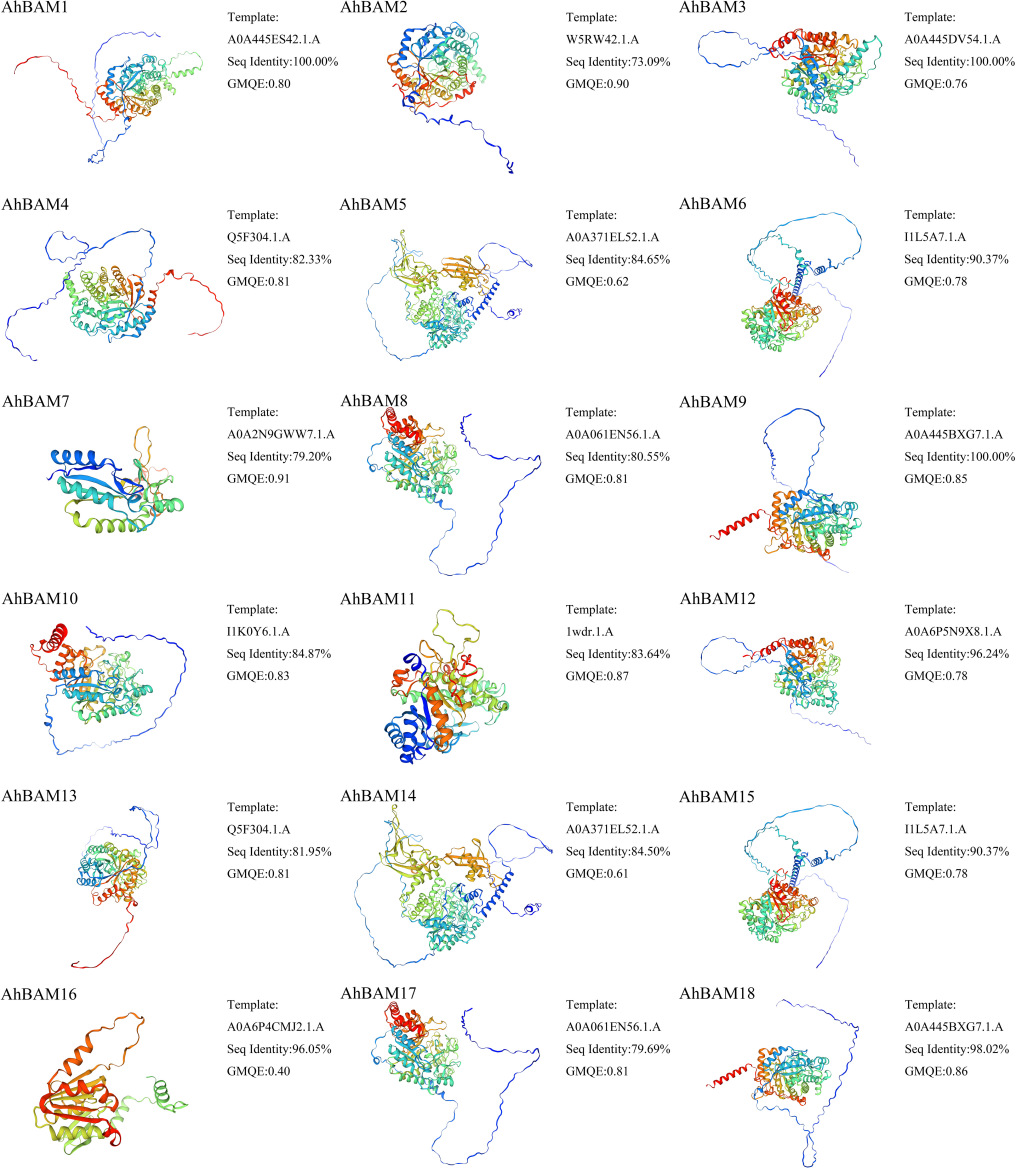
Three-dimensional structure prediction of AhBAM proteins.

**Figure 10:**
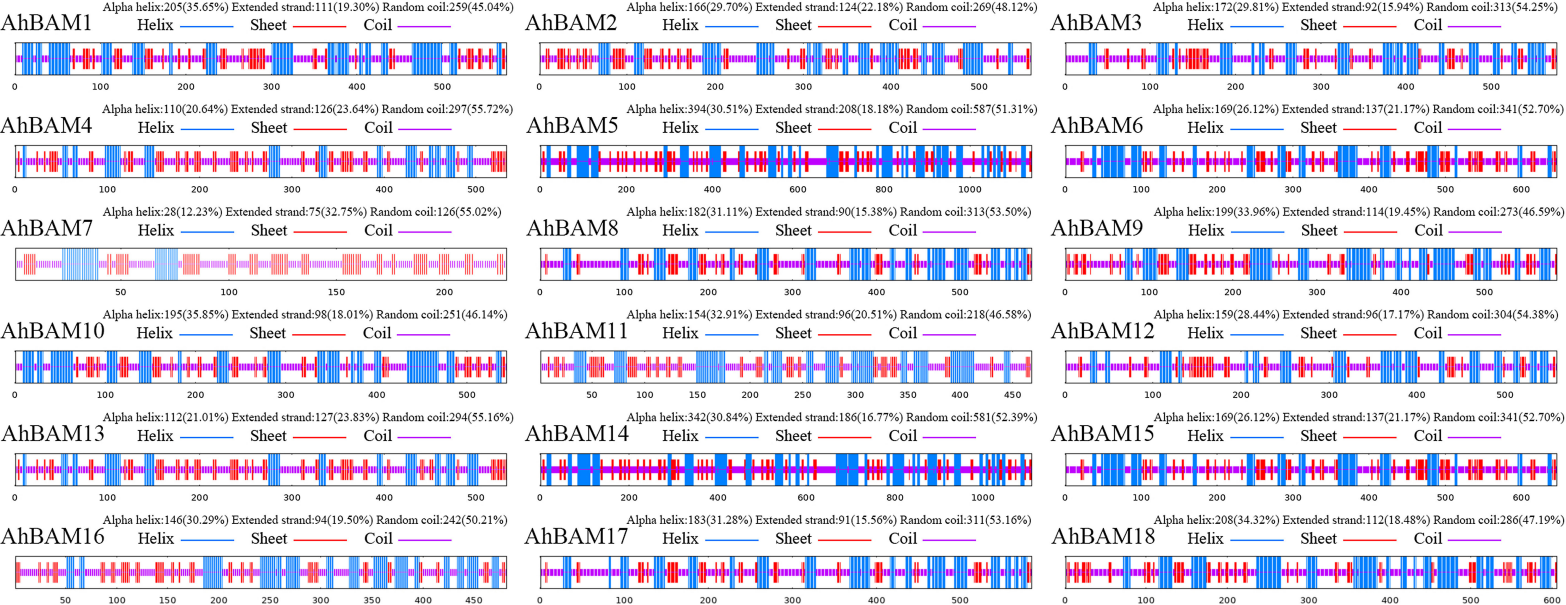
Secondary structure prediction of AhBAM proteins.

Predicted three-dimensional (3D) structures of AhBAM proteins demonstrated sequence identity values ranging from 73.09% to 100% (**Figure 9**). AhBAM1, AhBAM3, and AhBAM9 exhibited the highest sequence identity, while AhBAM2 showed the lowest conservation. The structural reliability, as assessed by global model quality estimation (GMQE) scores, ranged from 0.40 to 0.91, indicating high-confidence predictions suitable for functional interpretation. Secondary structure analysis confirmed the critical structural role of α-helices (right-handed coiled structures stabilized by backbone hydrogen bonds) in determining protein conformation and function. The predicted α-helices composition ranged from 12.23% to 35.85%, suggesting these secondary structure elements may contribute to functional specialization through their impact on protein folding and domain architecture (**Figure 10**).

### Expression pattern of *AhBAM* family members

**Figure 11:**
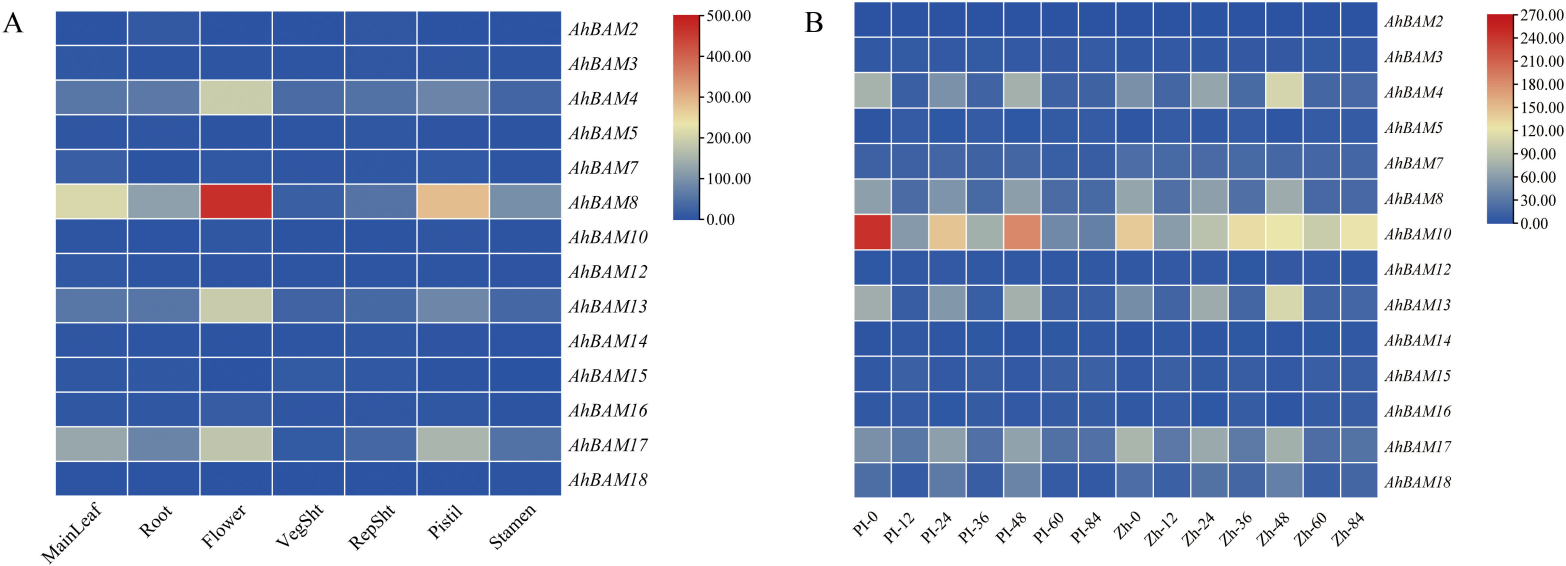
Expression profiles analysis of the peanut *BAM* gene family. **(A)** Heatmap of *AhBAM* gene expression in different peanut tissues. **(B)** Expression patterns of *AhBAM* genes under web blotch infection, as determined by RNA-seq.

Our research of *AhBAM* gene expression in seven peanut tissues (leaves, roots, flowers, branches, reproductive buds, pistils, and stamens) revealed distinct tissue-specific expression profiles (**Figure 11A**). Specifically, *AhBAM8* and *AhBAM18* exhibited higher expression in leaves, while *AhBAM4*, *AhBAM8*, *AhBAM13*, and *AhBAM17* were preferentially expressed in flowers. *AhBAM8* and *AhBAM17* exhibited particularly high transcript levels in pistils. In contrast, other tissues maintained relatively low expression of *AhBAM* genes (**Figure 11A**). The consistent high expression of *AhBAM8* in multiple tissues (leaves, flowers, and pistils) revealed its fundamental role in both vegetative and reproductive development of peanut.

Based on previous study, we performed the analysis of *AhBAM* gene expression in response to web blotch disease in peanuts. Results indicated that most *AhBAM* genes exhibited no significant transcriptional changes upon pathogen infection, with the exception of AhBAM10, which showed moderately elevated expression (**Figure 11B**). These findings suggest that *AhBAM* genes are unlikely to play a major role in the defense response against web blotch disease, though the specific function of *AhBAM10* needs further investigation.

**Figure 12:**
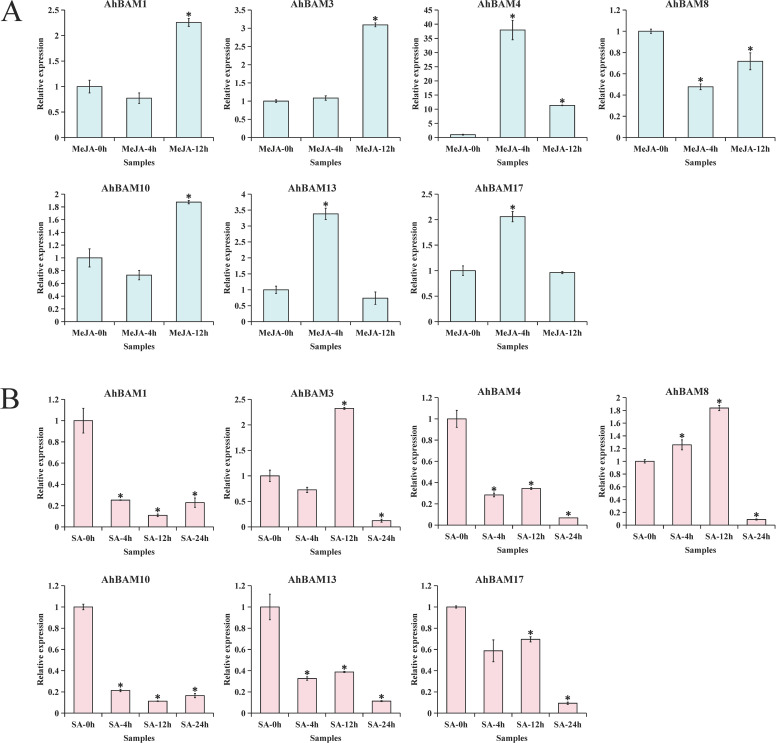
qRT-PCR validation of 7 *AhBAM* candidate genes induced by MeJA **(A)** and SA **(B)**. * indicates statistical significance at p < 0.05.

Our screening of publicly available expression datasets revealed seven AhBAM genes exhibiting putative stress-response characteristics. To investigate the roles of these genes in plant stress resistance mechanisms, we examined their dynamic expression patterns under treatments with core stress hormones (MeJA and SA). The results demonstrated that different AhBAM genes exhibited distinct temporal response patterns to hormonal stimuli: in the MeJA treatment group, AhBAM4, AhBAM13, and AhBAM17 functioned as early-response genes (showing >2-fold upregulation at 4 hours), while AhBAM1, AhBAM3, and AhBAM10 displayed late-response characteristics (>2-fold upregulation at 12 hours) (**Figure 12A**). However, SA treatment induced a different regulatory pattern: except for AhBAM3 which showed specific upregulation at 12 hours, four genes including AhBAM1 and AhBAM4 exhibited significant downregulation at the early stage (4 hours) (**Figure 12B**). These results demonstrate distinct functional specialization of AhBAM genes in phytohormone responses, with AhBAM3 emerging as a particularly noteworthy candidate due to its putative role in cross-talk between multiple stress response mechanisms.

## Discussion

### Genome-wide identification of the *BAM* gene family in different species

β-Amylases are evolutionarily conserved enzymes that catalyze the production of maltose and participate in plant metabolism and stress responses (Thalmann et al., 2019). The *BAM* gene family exhibits significant subfamily differentiation and functional diversity during evolution. Studies have shown that *BAM* genes in upland cotton, (27 *GhBAM* genes, 3 subfamilies) and jujube (9 *BAM* genes, 4 subfamilies), primarily regulate basic metabolic processes (Yang et al., 2023; Ma et al., 2022). Whereas those in pomegranate (8 *PgBAM* genes) and Chinese white pear (17 *PbBAM* genes) have evolved distinct stress-responsive functions, particularly in adaptation to cold and drought (Liu et al., 2024; Zhao et al., 2019). In this study, we identified 18 *AhBAM* genes in peanut, and classified into four subfamilies,. showing high consistency with species such as jujube and pomegranate. Futurermore, *AhBAM5* and *AhBAM14* from the G1 subfamily contain more than 24 introns, displaying complex structures similar to the stress-responsive *PbBAM* genes in pear. In contrast, *AhBAM4*, *AhBAM7*, and *AhBAM13* from the G3 subfamily possess only two introns, exhibiting a streamlined structure comparable to the *GhBAM* genes in cotton (Elsanosi et al., 2024). This cross-species structural-functional conservation suggests that peanut *BAM* genes may play dual roles in metabolic regulation and stress responses. Future research should focus on elucidating the molecular mechanisms of peanut *BAM* genes in abiotic stress responses, particularly the stress-adaptive functions of G1 subfamily genes, which could provide valuable candidate gene resources for breeding stress-resistant peanut varieties.

### Gene duplication and selection pressure driving *AhBAM* family expansion

Gene duplication, including small-scale duplications (tandem and segmental duplications), serves as a crucial driving force in eukaryotic genome evolution (Kuzmin et al., 2022). In the peanut, *AhBAM* gene family, we identified eight segmentally duplicated gene pairs in the peanut *AhBAM* gene family, while no tandem duplications were detected. These findings in soybean and other leguminous plants may represent the primary mechanism for the expansion of the *BAM* gene family in legumes. Additionally, selection pressure analysis revealed that these duplicated gene pairs have undergone strong evolutionary selection, indicating their essential role in maintaining the conserved functions of *BAM* enzymes. Furthermore, synteny analysis between peanut and wild diploid relatives, as well as comparisons with other species, identified 34 syntenic gene pairs between peanut and soybean, a number significantly higher than that observed between peanut and other species. This not only confirmed their close evolutionary relationship but also provides new insights into the adaptive evolution of leguminous plants. These conserved duplicated gene pairs in peanut and soybean may have driven functional diversification of *BAM* genes through processes such as subfunctionalization, thereby conferring species-specific metabolic regulatory features.

### Functional diversity of the *BAM* gene family in peanuts

A comprehensive analysis of the *AhBAM* gene family in peanuts reveals its potential functions in plant growth, stress response, and metabolic regulation. Promoter analysis demonstrated that *AhBAM* genes are enriched with light-responsive (e.g., Box4), hormones-responsive, and abiotic stress-related cis-elements. This characteristic aligns with the regulatory patterns of *BAM* genes in tomato and sweet potato (Li et al., 2024; Huang et al., 2021), indicating conserved functions of this family in the crosstalk between light signaling, hormone responses, and stress adaptation. AhBAM3, positioned as a hub node in the protein-protein interaction network, likely integrates stress signals (e.g., SA and JA) with starch metabolic pathways to coordinate carbon allocation and energy homeostasis. This central regulatory feature closely resembles the function of Arabidopsis AtBAM3, suggesting that AhBAM3 may play a similarly critical role in peanut stress responses. These findings suggest that AhBAM3 subfamily members may have evolved analogous stress adaptation strategies in both leguminous and solanaceous plants during evolution, making them valuable molecular targets for improving stress resistance in crops. Exploring the regulatory mechanism of AhBAM3 could provide new insights into metabolic reprogramming and stress signaling in peanuts, offering potential applications in breeding stress-resilient varieties.

### The three-dimensional structure directly influences the function of BAM genes

Comprehensive analysis of PTMs and structural features in the AhBAM protein family reveals critical insights into their regulatory mechanisms and functional diversity in peanut. The abundant tyrosine phosphorylation in AhBAM1 and AhBAM5 may regulate their enzyme activity and protein-protein interactions. Additionally, this mechanism is particularly relevant to stress tolerance in peanuts. Moreover, the identification of acetylation and methylation in multiple AhBAMs further underscores the regulatory complexity of this family, as these PTMs can alter protein stability and subcellular localization, ultimately influencing starch degradation efficiency during seed development or stress adaptation (Yang et al., 2025). Structurally, the conserved sequence motifs and predicted three-dimensional folds highlight both functional conservation and diversity within the AhBAM family (Foerderer et al., 2022). The high similarity among AhBAM1, AhBAM3, and AhBAM9 suggests similarity function in maintaining basal starch metabolism, whereas the distinct features of AhBAM2) may reflect specialized functions, such as tissue-specific expression. The PTM-driven regulation of AhBAMs could be exploited to enhance stress resilience or starch accumulation in peanut seeds. Future studies should validate these modifications in planta, particularly under field-relevant conditions, to harness their potential for peanut breeding programs.

### *AhBAM* is likely to possess multiple biological functions, particularly in responding to environmental stimuli

Transcriptome data analysis revealed distinct spatial regulation of peanut *AhBAM*, with *AhBAM8* exhibited high expression in photosynthetic (main stem leaves) and reproductive tissues (flowers and pistils), while *AhBAM4*/*13*/*17* showed flower-specific expression patterns. This tissue-specific partitioning strongly implicates certain *AhBAM* isoforms in floral development and reproductive processes. Peanut web blotch disease can occur throughout the entire growth period, often causing extensive leaf drop and severely affecting peanut yield and quality (Zhang et al., 2023). Most *AhBAM* genes showed no significant response to web blotch disease, except for *AhBAM10*, which exhibited moderate upregulation indicates that AhBAM-mediated starch metabolism may not be a primary defense mechanism against this pathogen. The distinct hormonal response patterns of AhBAM genes further emphasize their functional diversification. The early upregulation of *AhBAM4*, *AhBAM13*, and *AhBAM17* under MeJA suggests their involvement in jasmonate-mediated stress responses, possibly linked to wound healing. In contrast, the late induction of *AhBAM1*, *AhBAM3*, and *AhBAM10* implies roles in prolonged stress adaptation, such as drought. Importantly, *AhBAM3* emerged as a unique candidate due to its SA-specific upregulation, hinting at its potential role in balancing JA-SA crosstalk. The downregulation of multiple *AhBAM* genes under SA at early stages may reflect a trade-off between growth and defense. The expression dynamics of *AhBAM* genes underscore their tissue-specific and hormone-regulated roles in peanut development and stress adaptation.

## Conclusions

This study comprehensively analyzed the *BAM* gene family in peanuts, identifying 18 members unevenly distributed across 10 chromosomes. Subcellular localization showed most members in the nucleus and chloroplasts. Phylogenetic analysis grouped them into four subgroups with conserved gene structures and motifs. Promoter analysis revealed enrichment of cis-acting elements related to light, hormones, and stress responses. Intraspecific collinearity identified eight gene pairs under purifying selection, while interspecific collinearity with soybean highlighted 18 gene pairs, indicating close evolutionary relationships. Network analysis pinpointed AhBAM3 as a central hub in signal integration. Gene Ontology analysis confirmed functional diversity, mainly in starch and glucose metabolism. Epigenetic and structural predictions showed both conservation and diversity within the family. Transcriptome data highlighted roles in flowering and MeJA/SA responses but not in web blotch disease resistance. These findings provide a solid foundation for further elucidating the molecular mechanisms and functional roles of the peanut *BAM* gene family.

## Data Availability

The datasets presented in this study can be found in online repositories. The names of the repository/repositories and accession number(s) can be found below: https://www.peanutbase.org/, arahy.3VMA9Y, arahy.NQ88PQ, arahy.U96YPL, arahy.I36EUT, arahy.UANN5Y, arahy.K0N155, arahy.68PBWJ, arahy.4Y2508, arahy.C3I2PC, arahy.34GL3D, arahy.0V89VS, arahy.ABG1ZW, arahy.60DV95, arahy.M409IX, arahy.Y0TSMC, arahy.JJTS0M, arahy.TP5BL7, arahy.Y6Z03A.

## References

[B1] Bailey T. L. Johnson J. Grant C. E. Noble W. S. (2015). The MEME suite. Nucleic Acids Res. 43, W39–W49. doi: 10.1093/nar/gkv416 25953851 PMC4489269

[B2] Chen C. Wu Y. Li J. Wang X. Zeng Z. Xu J.. (2023a). TBtools-II: A “one for all, all for one”bioinformatics platform for biological big-data mining. Mol. Plant 16, 1733–1742. doi: 10.1016/j.molp.2023.09.010 37740491

[B3] Chen M. Zhang W. Gou Y. Xu D. Wei Y. Liu D.. (2023b). GPS 6.0: an updated server for prediction of kinase-specific phosphorylation sites in proteins. Nucleic Acids Res. 51, W243–W250. doi: 10.1093/nar/gkad383 37158278 PMC10320111

[B4] Chen Y. Yao Z. Sun Y. Wang E. Tian C. Sun Y.. (2022). Current studies of the effects of drought stress on root exudates and rhizosphere microbiomes of crop plant species. Int. J. Of Mol. Sci. 23, 2374. doi: 10.3390/ijms23042374 35216487 PMC8874553

[B5] Clevenger J. Chu Y. Scheffler B. Ozias-Akins P. (2016). A developmental transcriptome map for allotetraploid arachis hypogaea. Front. Plant Sci. 7. doi: 10.3389/fpls.2016.01446 PMC5043296 27746793

[B6] David L. C. Lee S. K. Bruderer E. Abt M. R. Fischer-Stettler M. Tschopp M. A.. (2022). BETA-AMYLASE9 is a plastidial nonenzymatic regulator of leaf starch degradation. Plant Physiol. 188, 191–207. doi: 10.1093/plphys/kiab468 34662400 PMC8774843

[B7] Elsanosi H. A. Zhang J. H. Mostafa S. Geng X. Y. Zhou G. S. Awdelseid A. H. M.. (2024). Genome-wide identification, structural and gene expression analysis of *BTB* gene family in soybean. BMC Plant Biol. 24, 663. doi: 10.1186/s12870-024-05365-1 38992596 PMC11238345

[B8] Foerderer A. Li E. T. Lawson A. W. Deng Y. N. Sun Y. Logemann E.. (2022). A wheat resistosome defines common principles of immune receptor channels. Nature 610, 532–539. doi: 10.1038/s41586-022-05231-w 36163289 PMC9581773

[B9] Horton P. Park K. J. Obayashi T. Fujita N. Harada H. Adams-Collier C. J.. (2007). WoLF PSORT: protein localization predictor. Nucleic Acids Res. 35, W585–W587. doi: 10.1093/nar/gkm259 17517783 PMC1933216

[B10] Huang X. F. Bi C. Y. Huang W. Q. Liu J. H. Hu Y. Z. Huang B. F.. (2021). Genome-wide identification and expression analysis of the β-amylase gene family in Ipomoea batatas. J. South China Agric. Univ. 42, 50–59. doi: 10.7671/j.issn.1001-411X.202011031

[B11] Huerta-Cepas J. Szklarczyk D. Heller D. Hernández-Plaza A. Forslund S. K. Cook H.. (2019). eggNOG 5.0: a hierarchical, functionally and phylogenetically annotated orthology resource based on 5090 organisms and 2502 viruses. Nucleic Acids Res. 47, D309–D314. doi: 10.1093/nar/gky1085 30418610 PMC6324079

[B12] Krejci A. Hupp T. R. Lexa M. Vojtesek B. Muller P. (2016). Hammock: a hidden Markov model-based peptide clustering algorithm to identify protein-interaction consensus motifs in large datasets. Bioinformatics 32, 9–16. doi: 10.1093/bioinformatics/btv522 26342231 PMC4681989

[B13] Krzywinski M. Schein J. Birol I. Connors J. Gascoyne R. Horsman D.. (2009). Circos: An information aesthetic for comparative genomics. Genome Res. 19, 1639–1645. doi: 10.1101/gr.092759.109 19541911 PMC2752132

[B14] Kuzmin E. Taylor J. S. Boone C. (2022). Retention of duplicated genes in evolution. Trends In Genet. 38, 59–72. doi: 10.1016/j.tig.2021.06.016 34294428 PMC8678172

[B15] Nguyen L. T. Schmidt H. A. von Haeseler A. Minh B. Q. (2015). IQ-TREE: A fast and effective stochastic algorithm for estimating maximum-likelihood phylogenies. Mol. Biol. And Evol. 32, 268–274. doi: 10.1093/molbev/msu300 25371430 PMC4271533

[B16] Lescot M. Dehais P. Thijs G. Marchal K. Moreau Y. Van de P.. (2002). PlantCARE, a database of plant cis-acting regulatory elements and a portal to tools for in silico analysis of promoter sequences. Nucleic Acids Res. 30, 325–327. doi: 10.1093/nar/30.1.325 11752327 PMC99092

[B17] Letunic I. Bork P. (2021). Interactive Tree Of Life (iTOL) v5: an online tool for phylogenetic tree display and annotation. Nucleic Acids Res. 49, W293–W296. doi: 10.1093/nar/gkab301 33885785 PMC8265157

[B18] Letunic I. Khedkar S. Bork P. (2021). SMART: recent updates, new developments and status in 2020. Nucleic Acids Res. 49, D458–D460. doi: 10.1093/nar/gkaa937 33104802 PMC7778883

[B19] Li X.. (2024). ‘Genome-wide identification of tomato (Solanum lycopersicum) β -BAM gene family and its expression analysis under salt stress and exogenous plant growth regulators. J. Agric. Biotechnol. 32, 1008–1019. doi: 10.3969/j.issn.1674-7968.2024.05.003

[B20] Liu L. B. Xu S. W. Zhang L. H. Zheng J. (2024). A genome-wide analysis of the *BAM* gene family and identification of the cold-responsive genes in pomegranate (*Punica granatum* L.). Plants-Basel 13, 1321. doi: 10.3390/plants13101321 38794392 PMC11125002

[B21] Lu S. N. Wang J. Y. Chitsaz F. Derbyshire M. K. Geer R. C. Gonzales N. R.. (2020). CDD/SPARCLE: the conserved domain database in 2020. Nucleic Acids Res. 48, D265–D268. doi: 10.1093/nar/gkz991 31777944 PMC6943070

[B22] Lu Q. Huang L. Liu H. Garg V. Gangurde S. Li H.. (2024). A genomic variation map provides insights into peanut diversity in China and associations with 28 agronomic traits. Nat. Genet. 56, 530–540. doi: 10.1038/s41588-024-01660-7 38378864

[B23] Lv Y. Yang M. Hu D. Yang Z. Y. Ma S. Q. Li X. H.. (2017). The OsMYB30 transcription factor suppresses cold tolerance by interacting with a JAZ protein and suppressing β-amylase expression. Plant Physiol. 173, 1475–1491. doi: 10.1104/pp.16.01725 28062835 PMC5291022

[B24] Ma Y. P. Han Y. R. Feng X. R. Gao H. D. Cao B. Song L. H. (2022). Genome-wide identification of *BAM* (β-amylase) gene family in jujube (*Ziziphus jujuba* Mill.) and expression in response to abiotic stress. BMC Genomics 23, 438. doi: 10.1186/s12864-022-08630-5 35698031 PMC9195466

[B25] Mistry J. Chuguransky S. Williams L. Qureshi M. Salazar G. A. Sonnhammer E. L. L.. (2021). Pfam: The protein families database in 2021. Nucleic Acids Res. 49, D412–D419. doi: 10.1093/nar/gkaa913 33125078 PMC7779014

[B26] Monroe J. D. Storm A. R. (2018). Review: The Arabidopsis β-amylase (BAM) gene family: Diversity of form and function. Plant Sci. 276, 163–170. doi: 10.1016/j.plantsci.2018.08.016 30348315

[B27] Niu L. J. Wu X. L. Liu H. Hu X. L. Wang W. (2024). Leaf starch degradation by β-amylase *ZmBAM8* influences drought tolerance in maize. Carbohydr Polym 345, 122555. doi: 10.1016/j.carbpol.2024.122555 39227118

[B28] Ravenburg C. M. Riney M. B. Monroe J. D. Berndsen C. E. (2022). The *BAM7* gene in *Zea mays* encodes a protein with similar structural and catalytic properties to *Arabidopsis* BAM2. Acta Crystallographica Section D-Structural Biol. 78, 560–570. doi: 10.1107/S2059798322002169 PMC9063847 35503205

[B29] Shannon P. Markiel A. Ozier O. Baliga N. S. Wang J. T. Ramage D.. (2003). Cytoscape: A software environment for integrated models of biomolecular interaction networks. Genome Res. 13, 2498–2504. doi: 10.1101/gr.1239303 14597658 PMC403769

[B30] Stajich J. E. (2007). An introduction to bioPerl. Methods Mol. Biol. (Clifton N.J.) 406, 535–548. doi: 10.1007/978-1-59745-535-0_26 18287711

[B31] Szklarczyk D. Kirsch R. Koutrouli M. Nastou K. Mehryary F. Hachilif R.. (2023). The STRING database in 2023: protein-protein association networks and functional enrichment analyses for any sequenced genome of interest. Nucleic Acids Res. 51, D638–D646. doi: 10.1093/nar/gkac1000 36370105 PMC9825434

[B32] Tang D. Chen M. Huang X. Zhang G. Zeng L. Zhang G.. (2023). SRplot: A free online platform for data visualization and graphing. PLoS One 18, e0294236. doi: 10.1371/journal.pone.0294236 37943830 PMC10635526

[B33] Thalmann M. Coiro M. Meier T. Wicker T. Zeeman S. C. Santelia D. (2019). The evolution of functional complexity within the -amylase gene family in land plants. BMC Evolutionary Biol. 19, 66. doi: 10.1186/s12862-019-1395-2 PMC6394054 30819112

[B34] Toomer O. T. (2018). Nutritional chemistry of the peanut (*Arachis hypogaea)* . Crit. Rev. In Food Sci. And Nutr. 58, 3042–3053. doi: 10.1080/10408398.2017.1339015 28662347

[B35] Wang D. Zhang Y. Zhang Z. Zhu J. Yu J. (2010). KaKs_Calculator 2.0: a toolkit incorporating gamma-series methods and sliding window strategies. Genomics Proteomics Bioinf. 8, 77–80. doi: 10.1016/S1672-0229(10)60008-3 PMC5054116 20451164

[B36] Wang Y. P. Tang H. B. DeBarry J. D. Tan X. Li J. P. Wang X. Y.. (2012). *MCScanX*: a toolkit for detection and evolutionary analysis of gene synteny and collinearity. Nucleic Acids Res. 40, e49. doi: 10.1093/nar/gkr1293 22217600 PMC3326336

[B37] Wang X. B. Sun Z. Q. Qi F. Y. Zhou Z. Y. Du P. Shi L.. (2025). A telomere-to-telomere genome assembly of the cultivated peanut. Mol. Plant 18, 5–8. doi: 10.1016/j.molp.2024.12.001 39628055

[B38] Waterhouse A. Bertoni M. Bienert S. Studer G. Tauriello G. Gumienny R.. (2018). SWISS-MODEL: homology modelling of protein structures and complexes. Nucleic Acids Res. 46, W296–W303. doi: 10.1093/nar/gky427 29788355 PMC6030848

[B39] Wu X. (2024). Fine mapping of resistant QTLs for peanut web blotch disease and study on the mechanisms of resistance respone. China: Henan Agricultural University. doi: 10.27117/d.cnki.ghenu.2024.000028

[B40] Xie Y. B. Li H. Q. Luo X. T. Li H. Y. Gao Q. Y. Zhang L. W. Y.. (2022). IBS 2.0: an upgraded illustrator for the visualization of biological sequences. Nucleic Acids Res. 50, W420–W426. doi: 10.1093/nar/gkac373 35580044 PMC9252815

[B41] Yang Y. L. Sun F. L. Wang P. L. Yusuyin M. Kuerban W. Lai C. X.. (2023). Genome-wide identification and preliminary functional analysis of *BAM* (β-amylase) gene family in upland cotton. Genes 14, 2077. doi: 10.3390/genes14112077 38003020 PMC10671626

[B42] Yang W. Feng M. Yu K. H. Cao J. Cui G. X. Zhang Y. M.. (2025). The TaCLE24b peptide signaling cascade modulates lateral root development and drought tolerance in wheat. Nat. Commun. 16, 1952. doi: 10.1038/s41467-025-57291-x 40000659 PMC11862134

[B43] Zhang X. G. Pandey M. K. Wang J. P. Zhao K. K. Ma X. L. Li Z. F.. (2021). Chromatin spatial organization of wild type and mutant peanuts reveals high-resolution genomic architecture and interaction alterations. Genome Biol. 22, 315. doi: 10.1186/s13059-021-02520-x 34784945 PMC8594070

[B44] Zhang M. Y. Tian M. D. Sun Z. Q. Qi F. Y. Wu X. H. Wang J.. (2023). Genetic analysis of resistance to web blotch in peanut. Chin. J. Oil Crop Sci. 45, 608–613. doi: 10.19802/j.issn.1007-9084.2022128

[B45] Zhang H. Tang Y. Y. Yue Y. L. Chen Y. (2024). Advances in the evolution research and genetic breeding of peanut. Gene 916, 148425. doi: 10.1016/j.gene.2024.148425 38575102

[B46] Zhao L. Y. Gong X. Gao J. Z. Dong H. Z. Zhang S. L. Tao S. T.. (2019). Transcriptomic and evolutionary analyses of white pear (*Pyrus bretschneideri*) β-amylase genes reveals their importance for cold and drought stress responses. Gene 689, 102–113. doi: 10.1016/j.gene.2018.11.092 30576803

